# Assessment of post‐SARS‐CoV‐2 fatigue among physicians working in COVID‐designated hospitals in Dhaka, Bangladesh

**DOI:** 10.1002/brb3.3553

**Published:** 2024-06-14

**Authors:** A. T. M. Hasibul Hasan, Nusrat Khan, Nazmul Hoque Munna, Wahidur Rahman Choton, Mostofa Kamal Arefin, Mohammad Abdullah Az Zubayer Khan, Mohaimen Mansur, Rashedul Hassan, Muhammad Shamsul Arefin, Nayema Afroze, Elham Nuzhat, Muhammad Sougatul Islam

**Affiliations:** ^1^ National Institute of Neurosciences & Hospital Dhaka Bangladesh; ^2^ BioTED Dhaka Bangladesh; ^3^ Mymensingh Medical College and Hospital Mymensingh Bangladesh; ^4^ Dhaka Medical College and Hospital Dhaka Bangladesh; ^5^ National Institute of Laboratory Medicine and Referral Center Dhaka Bangladesh; ^6^ Institute of Statistical Research and Training University of Dhaka Dhaka Bangladesh; ^7^ Department of Medicine Green Life Medical College & Hospital Dhanmondi Bangladesh; ^8^ 250 Bed District Hospital Kishoreganj Bangladesh; ^9^ Sir Salimullah Medical College and Mitford Hospital Dhaka Bangladesh

**Keywords:** fatigue, fatigue severity scale, post COVID

## Abstract

**Background:**

Fatigue has been observed after the outbreaks of several infectious diseases around the world. To explore the fatigue level among physicians working in COVID‐19‐designated hospitals in Bangladesh, a matched case‒control study was conducted on post‐SARS‐CoV‐2 fatigue.

**Method:**

In this study, 105 physicians diagnosed with COVID‐19 who were declared cured at least 6 weeks before the interview date were recruited as cases, and the same number of age‐ and designation‐matched healthy physicians were recruited as controls from the same hospital at a 1:1 ratio. Diagnosis of COVID‐19 infection was confirmed by detection of SARS CoV‐2 antigen by RT‒PCR from reference laboratories in Bangladesh or by HRCT chest.

**Result:**

Approximately two‐thirds of the physicians were male (67.6% vs. 32.4%). More than 80% of them were younger than 40 years. The cases had a significantly greater number of comorbid conditions. The fatigue severity scale (FSS) score (mean) was much higher for cases (36.7 ± 5.3 vs. 19.3 ± 3.8) than for the control group, with a statistically significant difference. Similarly, approximately 67.7% of the previously COVID‐19‐positive physicians were in the highest FSS score tertile compared to the respondents in the control group, who had a mean score of <3.

**Conclusion:**

Physicians who had a previous history of COVID‐19 infection had significantly higher total and mean FSS scores, signifying a more severe level of fatigue than physicians who had never been COVID‐19 positive while working in the same hospital irrespective of their age and sex.

## Graphical abstract

Physicians affected with COVID‐19 infection had higher level of fatigue than their colleagues.

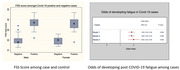

## 1 INTRODUCTION

COVID‐19 has created an unprecedented public health challenge globally since its emergence from Wuhan, China, in December 2019 (Zhu et al., 2019). The alarming rate of infectivity, considerable rate of mortality, and privation of effective treatment as well as an effective vaccine to prevent transmission have made the disease the greatest health and economic threat of the century (Fiani et al., 2020). Although initially emerged as a respiratory illness, COVID‐19 was later established to cause more widespread symptomatology due to its affinity for ACE‐2 receptors (Carod Artal, 2020). The first‐ever COVID‐19 case was detected in Bangladesh on March 8, 2020. Initially, the number of cases detected was scarce. However, with the sharp rise in case numbers, Bangladesh exceeded more than 2 million cases and nearly 30,000 COVID‐19‐related deaths by September 2023 (Who, 2021).

Presenting symptoms in the Bangladeshi population were commonly fever, cough, headache, myalgia, sore throat, malaise, and respiratory distress, similar to all affected countries in the world (Ahmed et al., 2020). Experience from recent pandemics of severe acute respiratory syndrome (SARS) and the Middle East respiratory syndrome (MERS) virus points to potential long‐term sequelae of COVID‐19 that are experienced worldwide (Fraser, 2021). Several studies have already suggested that COVID‐19 might not only leave a scar in the lung but also have a wide range of sequelae involving the cardiovascular and neuropsychiatric systems in terms of health (Ojha et al., 2020; Vindegaard & Benros, 2020).

In terms of mental health conditions, various studies have revealed a large number of cases of depression, anxiety, fatigue, and posttraumatic stress post SARS CoV‐19 and MERS (Vindegaard & Benros, 2020). Lee et al. (2007) observed that more than one‐third of hospitalized COVID‐19 patients suffered from moderate to severe symptoms of depression and anxiety. In addition to the general population, the postepidemic mental health conditions of healthcare workers and doctors have also been documented in various studies. Moldofsky and Patcai (2011) reported a syndrome of chronic fatigue, pain, weakness, depression, and sleep disturbance, which overlaps with the clinical and sleep features of fibromyalgia syndrome and chronic fatigue syndrome among healthcare workers after SARS CoV‐1 in a case‒control study.

Healthcare providers are also particularly vulnerable to emotional distress during the COVID‐19 pandemic, given their risk of exposure to the virus, concern about infecting and caring for their loved ones, shortages of personal protective equipment, longer working hours, and involvement in emotionally and ethically fraught resource‐allocation decisions (Pfefferbaum & North, 2020). A recent study on Chinese doctors and nurses reported that most of the doctors and nurses working in COVID‐19‐designated departments have a very high prevalence of psychological distress, anxious symptoms, and depressive symptoms (Liu et al., 2020). Long working hours, along with all these factors, can lead to fatigue, especially burnout. For Bangladesh, the prevalence of anxiety symptoms and depressive symptoms was 33.7% and 57.9%, respectively, and 59.7% had mild to extremely severe levels of stress among the general population of Bangladesh during the COVID‐19 pandemic (Banna et al., 2020). Until now, there has been no evidence on the mental health status of healthcare workers in Bangladesh during the pandemic, which can further affect their personal lives and professional commitments if they are not provided sufficient attention and required mental health services.

Therefore, we conducted this case‒control study to assess the level of fatigue after COVID‐19 infection among physicians working in a similar working environment in major COVID‐19‐designated hospitals in Bangladesh and to generate evidence on this unexplored scenario.

## 2 METHODOLOGY

A matched case‒control study was conducted between April 2020 and September 2020 among physicians working in 10 COVID‐19‐designated hospitals (both public and private) in Dhaka, Bangladesh. Data were collected from working physicians at Dhaka Medical College Hospital, Mugda Medical College Hospital, Kurmitola General Hospital, Kuwait Maitree Hospital, Mymensingh Medical College Hospital, Sayed Nazrul Islam Medical College Hospital, Green Life Medical College, Holy Family Red Crescent Medical College Hospital, and Anwar Khan Modern Medical College Hospital through a structured questionnaire.

### 2.1 Diagnosis and confirmation of SARS CoV‐2 infection

Diagnosis of SARS CoV‐2 infection was based on clinical presentation along with evidence of the presence of viral antigen by RT‒PCR test through the throat and nasal swab, irrespective of any evidence of COVID‐associated pneumonia in HRCT chest.

### 2.2 Case definition

Physicians, working either in a public or private COVID‐19‐designated hospital, who had a history of COVID‐19 infection as evidenced both by clinical features and RT‒PCR for SARS CoV‐2 antigen from nasal and throat swabs with or without HRCT chest and were declared cured by a negative RT‒PCR test result at least 6 weeks before the interview date were defined as a case for this study.

Physicians who were asymptomatic, but tested positive for COVID‐19 by RT‒PCR; were not included in this study. Furthermore, physicians who had typical HRCT changes for COVID‐19 infection but had negative RT‒ were also not included in this study.

### 2.3 Control definition

In this study, controls were healthy physicians, selected in a 1:1 ratio from each of the study centers matching for both age and professional designation with the cases, who had a negative report on RT‒PCR for SARS CoV‐2 infection by nasal and throat swab. All physicians working in COVID‐19‐designated hospitals are subjected to routine RT‒PCR tests for SARS CoV‐2 before and after their rotational ward duties.

### 2.4 Sample size

This was a matched case‒control study. Participants were matched based on age and their professional hierarchy (to correct confounding for exposure level, as it can be widely varied due to assigned duties). Matching was conducted on a 1:1 basis. As COVID‐19 is a newly emerging clinical condition and therefore inadequate data for prevalence or odds ratios to calculate the exact required sample size were not available, we adopted the traditional arbitrary method. Hence, an expected proportion among controls of 0.05%, an assumed odds ratio of 4, a 95% confidence interval, and 80% power, the required minimum sample size was 196 in total and 98 in each group. We increased the sample size to 210 with 105 participants each in the case and control groups.

#### 2.4.1 Study site and sampling

Data were collected from six government and four private hospitals in Dhaka city, all of which were dedicated to COVID‐19. A convenience sampling method was adopted to collect data for this study.

#### 2.4.2 Assessment of fatigue—FSS questionnaire

Fatigue can be defined as a subjective experience and includes symptoms such as rapid inanition, persisting lack of energy, exhaustion, physical and mental tiredness, and apathy (Chaudhuri & Behan, 2004). The level of fatigue was assessed using the fatigue severity scale (FSS), which has prior validation through various studies (Valko et al., 2008) and was developed by Krupp in 1989 (Armutlu et al., 2007). The FSS is a nine‐item questionnaire for the assessment of the severity of fatigue (Supporting Information Appendix). In this self‐administered questionnaire, the grading of each item ranges from 1–7, where 1 is designated for strong disagreement and 7 for strong agreement. The lowest score is 9 and the highest score is 63. The mean score of all items is also considered according to reporting/study methodology (Armutlu et al., 2007).

### 2.5 Data collection and analysis

Data were collected through a structured questionnaire including questions related to the basic demographic characteristics of the respondents, COVID‐19‐related status, and individual fatigue assessment. The data collection tool was pretested and modified before actual data collection. Data were collected by the researchers themselves. Data analysis was performed using STATA version 16. Both descriptive and inferential statistical tests were performed for data analysis and presentation. There were no missing data in the dataset. The mean FSS score and FSS score (total) were compared among cases and controls to see any difference in the level of fatigue between the two groups by *t*‐tests. Multivariate logistic regression models were used to establish any possible association. The *p*‐value to denote the level of significance was <.005 with Bonferroni correction as a post hoc test.

## 3 RESULT

In this study, a total of 210 age‐ and designation‐matched COVID‐19‐positive and COVID‐19‐negative physicians were interviewed at a 1:1 ratio. Approximately two‐thirds of the physicians were male (67.6% vs. 32.4% female). The majority were younger than 40 years (80.5%). Although 74.5% of them had some form of postgraduate degree, most of them were junior physicians working as medical officers or assistant registrars (82.9%) (Table 1). Most of the participants worked in different government hospitals, with an overall mean of 8.1 (SD 5.4) years. Approximately 77.1% of the previously COVID‐19‐positive physicians (cases) were aged below 40 years, whereas 83.8% in the COVID‐19‐negative group were of the same age. The differences in age, sex, and postgraduation degrees were statistically nonsignificant between the case and controls. However, for the number of preexisting comorbidities, the difference was statistically significant. The FSS score was much higher in cases than in the control group. The mean value of the FSS score was also much higher and statistically significant in the case group (36.7 vs. 19.3).

**TABLE 1:** Study participant description.

Variables	Case		Control		*p*‐value^a^
	*n*	%	*n*	%	
**Age (years)**					.012
<30	17	16.2	17	16.2	
31–40	67	63.8	67	63.8	
41–50	20	19	20	19	
51–60	1	1	1	1	
**Sex**					.140
Male	66	46.5	39	57.4	
Female	76	53.5	29	42.7	
**Postgraduate degree**					.004
None	16	29.6	38	70.4	
Diploma/MCPS/MPhil	23	51.1	22	48.9	
MD/MS	36	61.0	23	39.0	
FCPS	30	57.7	22	42.3	
**Current designation**					
Medical officer/assistant registrar/lecturer/registrar	87	82.9	87	82.9	
Junior consultant/assistant professor	15	14.3	15	14.3	
Senior consultant/associate professor	2	1.9	2	1.9	
Professor	1	0.9	1	0.9	
**Workplace**					.000
Public hospital/institute/medical college/university	51	76.1	16	23.9	
Private hospital/institute/medical college/clinic	54	37.8	89	62.4	
**Comorbidity**					.002
0	48	45.7	50	47.6	
1	27	25.7	14	13.3	
2–5	26	24.8	21	20.0	
>6	4	3.8	20	19.1	
**FSS score**					.000
1 (lowest)	1	1.4	72	98.6	
2	38	53.5	33	46.5	
3 (highest)	66	100.0	0	0.0	
	Mean	SD	Mean	SD	
**FSS score**	36.7	5.3	19.3	3.8	.000
**Years of the clinical career**	8.4	5.7	7.9	5.2	.5104

Abbreviation: FSS, fatigue severity scale.

^a^ Significance after Bonferroni correction.

**FIGURE 1:**
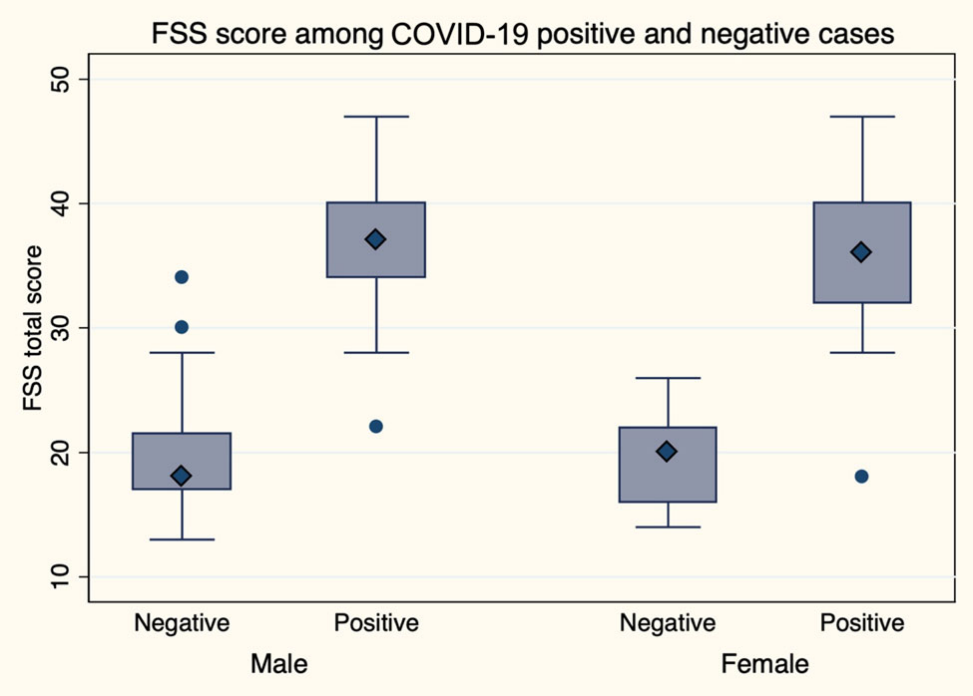
Fatigue severity scale (FSS) score among cases and controls.

In Table 2, gender differentials for various variables in COVID‐19‐positive participants are shown. Most of the participants were confirmed as COVID‐19 positive cases by the RT‒PCR test. Only one‐third of the participants required hospitalization, with a male majority. Only six of the total cases required ICU support. In terms of self‐perceived severity, more than 90% of cases were mild to moderate. Preexisting comorbidities were higher in men, but none of these characteristics were statistically significant. The relative FSS score was higher among the male cases, although the mean score was similar in males and females but statistically nonsignificant (Figure 1).

**TABLE 2:** COVID‐19‐related characteristics of the study participants.

Characteristics	Men		Women		Overall		*p*‐value
**COVID‐19 infection history**	*n*	%	*n*	%	*n*	%	
Positive	66	46.5	39	57.4	105	50.0	.140
Negative	76	53.5	29	42.7	105	50.0	
**Method of diagnosis**							.805
RT PCR	60	90.9	36	92.3	96	91.4	
RT PCR, HRCT Chest	6	9.1	3	7.7	9	8.6	
**Hospitalization**							
Yes	25	37.9	13	33.3	38	36.2	.640
No	41	62.1	26	66.7	67	63.8	
**ICU support**							.842
Yes	4	6.1	2	5.1	6	5.7	
No	62	93.9	37	94.9	99	94.3	
**Disease severity**							.655
Mild	26	39.4	18	46.2	44	41.9	
Moderate	36	54.6	18	46.2	54	51.4	
Severe	3	4.6	3	7.7	6	5.7	
Critical	1	1.5	0	0.0	1	1.0	
**Comorbidity**							.200
0	26	39.4	22	59.4	48	45.7	
1	19	28.8	8	20.5	27	20.5	
2–5	17	25.8	9	23.1	26	23.1	
>6	4	6.1	0	0.0	4	3.8	
**FSS score**							.180
1 (lowest)	0	0.0	1	2.6	1	2.6	
2	21	31.8	17	43.6	38	43.6	
3 (highest)	45	68.2	21	53.9	21	53.9	
**Postgraduate degree**							.524
None	10	15.2	6	15.4	16	15.2	
Diploma/MCPS/MPhil	13	19.7	10	25.6	23	21.9	
MD/MS	26	39.4	10	25.6	36	34.3	
FCPS	17	25.8	13	33.3	30	28.6	
**Current designation**							.418
Medical officer/assistant registrar/lecturer/registrar	52	78.8	35	89.7	87	82.9	
Junior consultant/assistant professor	11	16.7	4	10.3	15	14.3	
Senior consultant/associate professor	2	3.0	0.0	0.0	2	1.9	
Professor	1	1.5	0.0	0.0	1	1.0	
**Workplace**							.982
Public hospital/institute/medical college/university	32	48.5	19	48.7	51	48.6	
Private hospital/institute/medical college/clinic	34	51.5	20	51.3	54	51.4	
	Mean	SD	Mean	SD	Mean	SD	
**FSS score**	37.1	5.0	36.1	5.7	36.7	5.3	.3314
**Duration of illness**	12.3	3.3	11.5	3.2	12.0	3.3	.1976
**Duration of hospital stay**	2.5	3.7	2.0	3.1	2.3	3.5	.4803
**Number of symptoms**	8.0	3.6	6.7	3.5	7.5	3.6	.0758

Abbreviation: FSS, fatigue severity scale.

**FIGURE 2:**
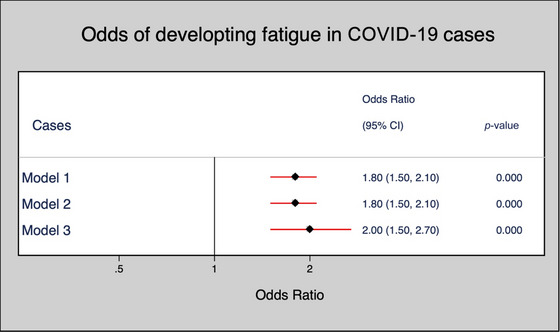
Odds of developing post‐COVID‐19 fatigue in the cases. CI, confidence interval.

Next, in the multivariate logistic regression, three models were implemented. There was no significant association between demographic and professional characteristics between cases and controls (Table 3). However, in terms of the FSS score, the odds of developing post‐COVID‐19 fatigue were two times higher in cases than in controls, which was significant in unadjusted, sex‐adjusted, and fully adjusted models (Figure 2).

**TABLE 3:** Multivariate analysis of predictor variables in cases compared with controls.

Characteristics	Model 1	Model 2	Model 3
**Sex**			
Male	1		1
Female	1.5 (0.8–2.8)		3.8 (0.6–23.3)
**Postgraduate degree**			
None	1	1	1
Diploma/MCPS/MPhil	2.5 (1.1–5.7)*	2.4 (1.1–5.6)*	0.4 (0.0–3.8)
MD/MS	3.7 (1.7–8.1)*	3.8 (1.7–8.3)*	2.3 (0.2–22.0)
FCPS	3.2 (1.5–7.2)	3.2 (1.4–7.1)*	0.1 (0.0–1.8)
**Workplace**			
Private hospital/institute/medical college/clinic	1	1	1
Public hospital/institute/medical college/university	5.3 (2.7–10.1)*	5.2 (2.7–10.1)*	6.7 (0.7–68.0)
**Comorbidity**			
No	1	1	1
Yes	1.1 (0.6–1.9)	1.1 (0.6–2.0)	0.7 (0.1–5.1)
**FSS score**	1.8 (1.5–2.1)*	1.8 (1.5–2.1)*	2.0 (1.5–2.7)*

*Note*: Model 1, unadjusted; Model 2, adjusted for sex; Model 3, fully adjusted.

Abbreviation: FSS, fatigue severity scale.

## 4 DISCUSSION

In this case‒control study of post‐COVID‐19 fatigue among physicians working in COVID‐19‐designated hospitals in Bangladesh, we reported a higher FSS score (both relative and mean) in previously COVID‐19‐positive physicians than in never‐positive physicians. However, we did not find evidence that there was any difference between cases and controls in terms of their sex, postgraduation degrees, number of years of service, or preexisting comorbid conditions. In fact, the difference in scores from cases indicated an increasing level of fatigue in physicians who had been positive for the virus infection and had returned to work.

Our initial literature review of major medical databases revealed a considerably low number of studies focusing on the mental health status of healthcare workers engaged directly in the diagnosis and treatment of patients infected with SARS CoV‐2. However, we found only one study that emphasized the mental health status of healthcare workers who previously contracted COVID‐19. Junhua et al. reported psychological factors and sleep status of medical staff contracting COVID‐19. In their case‒control study, they assessed the mental health status of 70 medical staff who had COVID‐19 (MEI et al., 2020). They used the Symptom Checklist‐90 (SCL‐90), Patient Health Questionnaire (PHQ‐15), Self‐rating Anxiety/Depression Scale (SAS/SDS), posttraumatic stress disorder self‐assessment scale (PTSD Checklist‐Civilian Version [PCL‐C]), and Pittsburgh Sleep Quality Index (PSQI) for the assessment of mental health. Those who were infected previously with COVID‐19 had significantly higher somatization, depression, anxiety, phobia, and sleep disturbances than the control group (MEI et al., 2020). This resonates with this understudied condition until now. Psychiatric comorbidities and persistent mental health problems have been reported in both short‐term and long‐term follow‐up studies after the pandemic of SARS CoV‐1 in 2003, (Lam, 2009; Lee et al., 2007; Tansey, 2007). Chronic fatigue syndrome has been reported in approximately 40.3% of survivors of SARS CoV‐1 (Lam, 2009). The strength of our study is that it is the first study in South Asia to explore this area of interest, and the finding of significantly high FSS scores in cases sets the basis for further exploration.

The long‐term effect of SARS‐CoV‐2 has yet to be established. Undue tiredness, irritability, sleep disturbance, fatigue, and other different somatic symptoms have been reported worldwide after previous outbreaks of various epidemics/pandemics. These are often termed postinfectious fatigue syndrome (PIFS) or postviral fatigue syndrome (Behan et al., 1985; Hickie et al., 2009, 2006; Naess et al., 2012).

The global prevalence of PIFS ranges from 0.2% to 0.42%, which has a substantial economic impact with the loss of productivity and loss of employment at the individual level (Jason et al., 2000; Lin et al., 2011; Nacul et al., 2011). The economic costs generated by chronic fatigue are high and mostly borne by patients and their families. Loss of income leads to a reduced standard of living, increases healthcare costs, and ultimately increases the expenditure budget on the respective government (Collin et al., 2011; Jason et al., 2008; Lloyd & Pender, 1994; Sabes‐Figuera et al., 2010).

Therefore, this study was conducted to understand the impact of COVID‐19 infection among physicians in Bangladesh working in COVID‐19‐designated hospitals. Inevitably, the majority of the physicians (80.5%) in this study were below 40 years of age and are the main driving force of the healthcare system of Bangladesh. Approximately two‐thirds of them were male physicians. Reasonably, the cases had a greater number of comorbidities. In light of our observation, Bajgain et al., 2021, in their review of 22 studies involving 22,753 COVID cases around the world, reported a higher prevalence of one or more comorbidities, including Cerebrovascular Disease (CVD) (8.9%), Hypertension (HTN) (27.4%), diabetes (17.4%), Chronic Obstructive Pulmonary Disease (COPD) (7.5%), cancer (3.5%), Chronic Kidney Disease (CKD) (2.6%), and other (15.5%).

The 9‐item FSS questionnaire was used for the assessment of fatigue among the case and control groups. The cases had a significantly higher (*p*‐value 0.000) mean FSS score (36.7 ± 5.3 vs. 19.3 ± 3.8) and a higher relative FSS score in higher tertiles. This denotes that although the physicians working under a similar stressful condition in COVID‐19‐designated hospitals irrespective of their age and designation, those with a previous history of contracting COVID‐19 infection had significantly more severe fatigue than their colleagues who had never been positive by RT‒PCR for SARS CoV‐2 in nasal and throat swab tests.

The mental health status of healthcare workers has been a great challenge since the outbreak of previous global pandemics; many times, they had a long‐term impact on the productivity and health status of the front liners (Chong et al., 2004; Wu et al., 2009). For COVID‐19, studies have documented that front‐line health workers directly involved with COVID‐19 patients had a higher risk of depression, anxiety, insomnia, and distress (Lai et al., 2020). Our findings with postinfection fatigue are similar. To reduce respondent bias and the influence of stress level at work, we compared the fatigue status of physicians working in different hospitals matching their designation. Additionally, the study was conducted at the peak stage of the pandemic; therefore, recall bias on the part of participants should be minimal.

The study is not free from limitations. First, there may be selection bias on relatively young physicians since the proportion of young physicians working in COVID‐19‐designated hospitals is much higher than that of seniors. We tried to correct this by age and designation matching. Moreover, senior physicians aged more than 50 are not directly involved in the management of COVID‐19 patients. The senior consultant physicians were supervising COVID‐19 management mostly online. Therefore, inferences cannot be drawn about the fatigue and comorbidity status of elderly/senior physicians. Various other confounding factors might have been unexplored, for example, environmental exposure, economic status, and family history. Finally, although case‒control study designs are efficient for rare diseases, a cohort study could yield multiple outcomes and stronger associations, which was not possible due to time and financial constraints.

## 5 CONCLUSION

Physicians who became COVID‐19‐positive while working at COVID‐19‐designated hospitals had a more severe level of fatigue at least 6 weeks post‐infection than their COVID‐negative colleagues working in the same hospital in Bangladesh. This is evident by a significantly higher relative and mean FSS score. This study highlights important insight into the less explored area of the mental health status of physicians and recommends further large‐scale studies to design interventions.

## AUTHOR CONTRIBUTIONS

**A. T. M. Hasibul Hasan**: Conceptualization; methodology; writing—original draft; visualization; supervision. **Nusrat Khan**: Methodology; formal analysis; supervision; writing—review and editing. **Nazmul Hoque Munna**: Data curation; writing—review and editing. **Wahidur Rahman Choton**: Investigation; data curation; writing—review and editing. **Mostofa Kamal Arefin**: Methodology; data curation; writing—review and editing. **Mohammad Abdullah Az Zubayer Khan**: Methodology; writing—review and editing; data curation. **Mohaimen Mansur**: Data curation; writing—review and editing; methodology; formal analysis. **Rashedul Hassan**: Writing—review and editing; data curation. **Muhammad Shamsul Arefin**: Data curation; writing—review and editing. **Nayema Afroze**: Writing—review and editing; data curation. **Elham Nuzhat**: Methodology; data curation; writing—review and editing. **Muhammad Sougatul Islam**: Methodology; visualization; writing—review and editing; data curation.

## FUNDING INFORMATION

The study received no funding, and neither of the authors received any financial support in the form of salaries for conducting the study.

## CONFLICT OF INTEREST STATEMENT

The authors declare no conflicts of interest.

### PEER REVIEW

The peer review history for this article is available at https://publons.com/publon/10.1002/brb3.3553

## Supporting information

Fatigue Severity Scale

## Data Availability

The data that support the findings of this study are openly available in MedRxIV at https://www.medrxiv.org/content/10.1101/2021.02.08.21251352v1, reference number https://doi.org/10.1101/2021.02.08.21251352.
