# Supplementary material for: Assessment of post‐SARS‐CoV‐2 fatigue among physicians working in COVID‐designated hospitals in Dhaka, Bangladesh
Source: Brain Behav. 2024 Jun 14;14(6):e3553. doi: 10.1002/brb3.3553 (PMC11177034; doi:10.1002/brb3.3553)
Supplement: Supplementary file 1 — Fatigue Severity Scale [file BRB3-14-e3553-s001.docx]

**Appendix**

Fatigue Severity Scale

| Fatigue Severity Scale (English) | | | | | | | | |
| --- | --- | --- | --- | --- | --- | --- | --- | --- |
|  | | | | | | | | |
|  |  | Strongly disagree Strongly agree | | | | | | |
|  |  |  | | | | | | |
| 1 | My motivation is lower when I am fatigued | 1 | 2 | 3 | 4 | 5 | 6 | 7 |
| 2 | Exercise brings on my fatigue | 0 | 0 | 0 | 0 | 0 | 0 | 0 |
| 3 | I am easily fatigued | 0 | 0 | 0 | 0 | 0 | 0 | 0 |
| 4 | Fatigue interferes with my physical functioning | 0 | 0 | 0 | 0 | 0 | 0 | 0 |
| 5 | Fatigue causes frequent problems for me | 0 | 0 | 0 | 0 | 0 | 0 | 0 |
| 6 | My fatigue prevents sustained physical functioning | 0 | 0 | 0 | 0 | 0 | 0 | 0 |
| 7 | Fatigue interferes with carrying out certain duties and responsibilities | 0 | 0 | 0 | 0 | 0 | 0 | 0 |
| 8 | Fatigue is among my three most disabling symptoms | 0 | 0 | 0 | 0 | 0 | 0 | 0 |
| 9 | Fatigue interferes with my work, family, or social life | 0 | 0 | 0 | 0 | 0 | 0 | 0 |
| Patients are instructed to choose a number from 1 to 7 that indicates their degree of agreement with each statement where 1 indicates strongly disagree and 7 strongly agree. | | | | | | | | |
